# Pheochromocytoma (PHEO) and Paraganglioma (PGL)

**DOI:** 10.3390/cancers11091391

**Published:** 2019-09-18

**Authors:** Karel Pacak, David Taïeb

**Affiliations:** 1 Section on Medical Neuroendocrinology, Head, Developmental Endocrine Oncology and Genetics Affinity Group. Eunice Kennedy Shriver NICHD, NIH, Building 10, CRC, Room 1E-3140, 10 Center Drive MSC-1109, Bethesda, MD 20892-1109, USA; 2 Department of Nuclear Medicine, La Timone University Hospital, European Center for Research in Medical Imaging, Aix-Marseille University, 13100 Marseille, France

This series of 23 articles (17 original articles, six reviews) is presented by international leaders in pheochromocytoma and paraganglioma (PPGL). PPGLs are rare neuroendocrine tumors originating from chromaffin cells in the adrenal medulla or paraganglia outside the adrenal medulla, respectively. Uniquely, these tumors produce and secrete catecholamines, mainly norepinephrine and epinephrine, that profoundly affect cardiovascular [1], gastrointestinal, and to lesser extents, other systems. One article shows that pheochromocytoma patients have a lower magnitude of global longitudinal strains (GLS) derived from speckle-tracking echocardiography compared to patients with essential hypertension, suggesting that catecholamines induce a subclinical decline in the left ventriclar systolic function [2]. Furthermore, if these tumors remain unrecognized, they pose a severe threat to patients by potentially causing sudden death due to lethal arrhythmias, myocardial infarction, and stroke. Therefore, all attempts should be made to diagnose and treat these tumors early before they strike a patient or become metastatic. Throughout the years, our knowledge and perception of these tumors have been greatly expanded and changed by new discoveries in genetics, metabolomics, proteomics, diagnostics, treatment, and follow-up of these tumors. Recently, there have been discoveries of new susceptible genes with either germline or somatic mutations [3]. Uniquely, metabolomic analysis has greatly improved the identification of these new genes and their pathogenicity, as well as the characterization of some variants of unknown significance. In this book, the spectrum of these new genes are described, as well as the implications on clinical management of patients. Recent studies have shown some gene-specific clinical risks that may warrant tailored management strategies [4]. The relevance of such mutations in tumorigenesis and catecholamine biosynthesis and secretion are also presented [5] with special emphasis on the role of hypoxia-inducible factors on the regulation of phosphorylation of tyrosine hydroxylase [6]. These findings, together with the excellent negative predictive value of histological PASS and GAPP algorithms [7], provide novel prognostic biomarkers and new therapeutic avenues [8,9,10]. Beyond catecholamines, PPGLs could also secrete a wide diversity of products which could serve as biomarkers, such as chromogranin A [11], and could be responsible in very exceptional situations of ectopic syndromes (mostly ACTH, IL6, PTH/PTHrp) [12]. A long-term overproduction of catecholamines by PPGL could also lead to the elevation of FGF21, especially in patients with secondary diabetes, that would require specific investigation to determine potential effects on metabolism and adipose tissue [13]. In recent years, molecular imaging has emerged at the forefront of personalized medicine. The use of molecular imaging, particularly with positron emission tomography compounds, in the localization of these tumors has been successfully expanded. Despite limited availability, [^11^C]-hydroxyephedrine PET/CT has shown to be an accurate tool to diagnose and rule out pheochromocytoma in complex clinical scenarios and to characterize equivocal adrenal incidentalomas [14]. More specifically for head and neck PGL and metastatic cases, [^68^Ga]-DOTATATE PET/CT has become the best available imaging modality. These results prompted the introduction of peptide receptor radionuclide therapy using radiolabeled somatostatin analogs. At present, more than 200 PPGL patients have been treated on compassionate grounds with PRRT with promising results. Here, Vyakaranam et al. [15] report a series of patients with favorable outcomes and limited toxicity. PET/CT or PET/MR imaging using a specific tracer such as [^18^F]-FDOPA might also allow improvement in treatment planning for external beam radiotherapy by allowing refinement of the gross tumor volume [16]. Kohlenberg et al. [17] also show excellent results of ablative therapy in the treatment of metastatic PPGL in order to achieve local control and decrease symptoms and signs from catecholamine excess. Given the potential for serious procedure-related complications, the balance-risk ratio should be discussed in each individual situation, and ablation procedures should be performed in high-volume centers. Throughout these therapies, as well as other situations (e.g., surgery), physicians must be aware of potential complications and be able to provide appropriate management to minimize morbidity and mortality associated with PPGLs, especially elevated catecholamine levels [18].

Although therapeutic and preventative options for PPGLs, especially metastatic disease, are still in their infancy, several new studies are now in progress or planned. To achieve these goals, preclinical models are needed, such as transgenic mice (e.g., Epas1 Gain-of-Function Mutation [19]), canine models that carry similar genomic alterations to humans [20], or patient-derived tumor xenografts (PDXs). This will accelerate our understanding on tumorigenesis, help to build original developmental models [21], and find new treatments. One promising approach in patients with metastatic PPGL relies on immunotherapy that initially activates innate immunity followed by an adaptive immune response. One original article shows a significant reduction or complete eradication of subcutaneous and metastatic lesions in a pheochromocytoma mouse model after immunotherapy using Mannan-BAM, TLR ligands, and anti-CD40 [22]. A pan-cancer RNA sequencing analysis also challenges the current classification of PPGL with clustering of PPGL with pancreatic neuroendocrine tumors or neuroblastomas, a finding that could open new therapeutic perspectives and help us understand the development of these tumors and their relationships [23]. The use of artificial intelligence, sophisticated computer algorithms, and modeling to classify information from a particular patient, as well as diagnostic and other methods done on that patient, will become a reality in the near future. This creates the potential to transform the lives of patients with these tumors, resulting in their prevention or even eradication. This series of unique articles represents a collaborative, international effort that reflects the scope and spirit of this issue by nicely blending current and future genetic, diagnostic, and therapeutic approaches to PPGLs. Understanding developmental, host, and environmental factors will also become very important to develop preventive strategies.

Let us conclude with a quotation from Dr. William Mayo: «The glory of medicine is that it is constantly moving forward, that there is always more to learn» Indeed, this issue provides new information not only to health care professionals but to basic scientists and others interested in learning something new about PPGL.
